# Silymarin Ameliorates Metabolic Dysfunction Associated with Diet-Induced Obesity via Activation of Farnesyl X Receptor

**DOI:** 10.3389/fphar.2016.00345

**Published:** 2016-09-28

**Authors:** Ming Gu, Ping Zhao, Jinwen Huang, Yuanyuan Zhao, Yahui Wang, Yin Li, Yifei Li, Shengjie Fan, Yue-Ming Ma, Qingchun Tong, Li Yang, Guang Ji, Cheng Huang

**Affiliations:** ^1^School of Pharmacy, Shanghai University of Traditional Chinese MedicineShanghai, China; ^2^School of Chemical and Environmental Engineering, Shanghai Institute of TechnologyShanghai, China; ^3^Brown Foundation Institute of Molecular Medicine and Program in Neuroscience, Graduate School of Biological Sciences, University of Texas McGovern Medical SchoolHouston, TX, USA; ^4^Research Centre for Traditional Chinese Medicine of Complexity Systems, Shanghai University of Traditional Chinese MedicineShanghai, China; ^5^Institute of Digestive Disease, Longhua Hospital, Shanghai University of Traditional Chinese MedicineShanghai, China

**Keywords:** silymarin, silybin, metabolic syndrome, non-alcoholic fatty liver disease, farnesyl X receptor

## Abstract

**Background and purpose:** Silymarin, a standardized extract of the milk thistle seeds, has been widely used to treat chronic hepatitis, cirrhosis, and other types of toxic liver damage. Despite increasing studies on the action of silymarin and its major active constituent, silybin in their therapeutic properties against insulin resistance, diabetes and hyperlipidaemia *in vitro* and *in vivo*, the mechanism underlying silymarin action remains unclear.

**Experimental approach:** C57BL/6 mice were fed high-fat diet (HFD) for 3 months to induce obesity, insulin resistance, hyperlipidaemia, and fatty liver. These mice were then continuously treated with HFD alone or mixed with silymarin at 40 mg/100 g for additional 6 weeks. Biochemical analysis was used to test the serum lipid and bile acid profiles. Farnesyl X receptor (FXR) and nuclear factor kappa B (NF-κB) transactivities were analyzed in liver using a gene reporter assay based on quantitative RT-PCR.

**Key results:** Silymarin treatment ameliorated insulin resistance, dyslipidaemia and inflammation, and reconstituted the bile acid pool in liver of diet-induced obesity. Associated with this, silybin and silymarin enhanced FXR transactivity. Consistently, in HepG2 cells, silybin inhibited NF-κB signaling, which was enhanced by FXR activation.

**Conclusion and implications:** Our results suggest that silybin is an effective component of silymarin for treating metabolic syndrome by stimulating FXR signaling.

## Introduction

Metabolic syndrome encompasses a cluster of conditions including obesity, type 2 diabetes, dyslipidaemia, atherosclerosis, cardiovascular disease, NAFLD and hepatobiliary diseases (Bonora, 2006; Rui, 2014). Because of rapid transition to over-nutritious environment and concealed disease progression, MS has become an epidemic and one leading killer worldwide (Finucane et al., 2011; Wahi et al., 2011). Maintaining a lifestyle with balanced energy metabolism is necessary to prevent but is not sufficient to treat MS, suggesting a critical importance for pharmacotherapy in treating and reversing MS. However, due to its complex pathogenesis, there are few approved prescription drugs that are able to effectively cure MS (Heal et al., 2009; Douglas et al., 2013).

The farnesyl X receptor (FXR, Nr1h4) serves as an intracellular BA-responsive ligand-activated NR (Makishima et al., 1999). It is ubiquitous in all tissues but highly expressed in liver, ileum, and adrenal gland (Matsubara et al., 2013). FXR has been shown to play an essential role in controlling BA homeostasis and protecting against liver injury resulting from cholestasis or drug induction, as well as maintaining normal lipid and glucose metabolism by regulating the expression of a series of downstream target genes (Westin et al., 2005; Cariou et al., 2006; Lee et al., 2006; Zhang et al., 2006, 2012; Kim et al., 2007; Matsubara et al., 2013). FXR^-/-^ mice have an array of metabolic derangements including impaired glucose tolerance and insulin sensitivity, increased plasma lipids levels, serious NAFLD and liver damage, etc. (Kong et al., 2008; Lefebvre and Staels, 2014). FXR is also reported to protect against toxic liver damage, hepatitis, and cirrhosis (Liu et al., 2003; Yang et al., 2007; Matsubara et al., 2013; Beuers et al., 2015). Targeting FXR is considered as a promising avenue in the treatment of liver diseases.

Inflammation is a complex biological response that is involved in various diseases. It has been reported that the FXR signal is involved in regulation of the inflammation response (Arrese and Karpen, 2010; Gadaleta et al., 2011). Antagonizing FXR can interact with NF-κB signaling leading to exacerbation of inflammation (Wang et al., 2008). NF-κB as a nuclear transcription factor directs the inflammatory response by controlling the expression of genes involved in inflammation (Hayden and Ghosh, 2008). In addition, numerous studies have implicated the NF-κB inflammatory program in the development of multiple metabolic diseases, and almost all liver diseases accompany hepatic inflammation (Elsharkawy and Mann, 2007). Interfering with NF-κB-driven inflammation could decrease hyperglycaemia and insulin resistance in patients (Baker et al., 2011).

In the US and Europe, about 65% of patients with liver metabolic disorders take herbal medicine (Tindle et al., 2005). Due to their apparent beneficial effects to human health, treatments with natural herbal medicine for diverse chronic MSs have become more and more popular (Stickel et al., 2002; Corns, 2003; Adewusi and Afolayan, 2010; Salomone et al., 2016). Silymarin is a natural herbal extraction from the fruit and seeds of the *Silybum marianum* (Milk thistle), consisting of seven flavonolignans (silybin A, silybin B, isosilybin A, isosilybin B, silychristin, isosilychristin, and silydianin; Kvasnička et al., 2003; Abenavoli et al., 2010; Biedermann et al., 2014). Milk thistle fruits and seeds have been used to treat liver and biliary disorders for more than 2000 years (Saller et al., 2001; Hoofnagle, 2005; Tamayo and Diamond, 2007; Abenavoli et al., 2010). Previous studies have proved that silymarin have antioxidant, anti-inflammation and anti-cancer effect properties, and improve homeostatic maintenance of glycaemia (Angulo et al., 2000; Kidd and Head, 2005; Gazák et al., 2007; Loguercio et al., 2012; Polyak et al., 2013). The flavonolignan silybin (a 1:1 mixture of silybin A and silybin B, which accounts for about 50–70% of the silymarin extract) is identified as the major active ingredient in silymarin (Kvasnička et al., 2003; Lee and Liu, 2003). Silymarin is used in the treatment of toxic liver damage, chronic hepatitis, and cirrhosis in some EU countries and China (Saller et al., 2001). Although clinical practices and animal experiments have repeatedly proven the effective therapeutic roles of silymarin and silybin *in vivo* (Tamayo and Diamond, 2007), the underlying molecular mechanism remains largely unknown. In the past 10 years, the mechanistic studies of silymarin and silybin were concentrated on reducing the mitochondrial ROS generation, inhibiting glycogenolysis and gluconeogenesis or blocking the activation of intrahepatic NF-κB signal pathway (Velussi et al., 1997; Abenavoli et al., 2010; Wu et al., 2011; Raina et al., 2013). No clear molecular target of silymarin or silybin has been reported. Here we identified silymarin and silybin as novel FXR agonists, and found that silybin inhibited the NF-κB signal pathway via negative crosstalk with the FXR in hepatocytes. In animal experiments, we found silymarin attenuated hyperlipidaemia, insulin resistance, and altered the composition of liver BAs pool in HF diet-fed C57BL/6 mice. These therapeutic effects of silymarin *in vivo* may be linked to the regulation of FXR and NF-κB signaling pathways by silybin, the major active constituent in silymarin.

## Materials and Methods

### Chemicals and Diets

Silymarin (Legalon, Madaus, German) powder was dissolved in dimethylsulfoxide to the final concentration of 40 mg/ml for cell culture. Silybin, GW4064, T090173, Rosiglitazone, int-777, WY14643, rifampicin, TNFα, and all of the BAs standard references mentioned in this article were purchased from Sigma–Aldrich (St. Louis, MO, USA). HF diets (60% of calories derived from fat), and low-calorie diets (10% of calories derived from fat) were purchased from Research Diet (D12492, D12450B, New Brunswick, NJ, USA).

### Cell Culture and Treatment

HepG2 (ATCC) cells were seeded on six-well plates (1 × 10^6^ cells/well) and grown to 80% confluence with high-glucose DMEM containing 10% FBS at 37°C in 5% CO_2_. The following day, cells were treated with vehicle control, silymarin (40 μg/ml) or silybin (25 μM). After 18-h treatment, the cells were treated with or without TNFα (10 ng/mL) and then collected for RNA isolation after 6-h incubation.

### Transient Transfection of Cultured Cells and Reporter Assays

The reporter assay was performed using the Dual-Luciferase Reporter Assay System (Promega, USA) as previously described (Huang et al., 2006). For NR transcription activity assay, the expression plasmids for phFXR, phRXR and FXR-dependent reporter (EcRE-LUC), pCMXGal-hPPARα, γ, LXRα, β, and PXR-LBD and the Gal4 reporter vector MH100 × 4-TK-Luc were co-transfected with a reporter construct so that 1 μg of the relevant plasmid combined with 1 μg of reporter plasmids and 0.1 μg of pREP7 (*Renilla luciferase*) reporter could be used to normalize transfection efficiencies. The transfection mixture, which contained 10 μg of total plasmids and 15 μl FuGENE-HD (Roche) per ml of DMEM, was added to HEK293T cells (ATCC) for 24 h and then removed. The FXR, PPARα, PPARγ, LXRs, and PXR agonists (GW4064, WY14643, Rosiglitazone, T090173, and Rifampicin, respectively), silymarin or silybin were added to fresh media and the cells were incubated for another 24 h to determine luciferase activity.

To characterize NF-κB activity, HepG2 cells were co-transfected with p65 expression vector, NF-κBx3-LUC, control plasmid pREP7, and/or FXR/RXR expression plasmids using FuGENE-HD. After 24 h, cells were pre-treated with the control, silymarin, or silybin for 18 h before treatment with TNFα (10 ng/mL) for 6 h. Subsequently, cells were collected and subjected to luciferase activity analysis. To determine the level of hTGR5 activation, hTGR5 expression plasmid and luciferase reporter pCRE-Luc and pREP7 were transiently transfected into HEK293T cells for 24 h. Then cells were incubated with the control, silymarin (40 μg/ml), or silybin (50 μM) for another day before being used for reporter assay. The renilla luciferase activity was assayed to normalize transfection efficiencies. All of the transfection experiments were performed in triplicate and repeated at least three times independently.

### Animal Experiment

All animal protocols used in this study were approved by Shanghai University of Traditional Chinese Medicine (Approval Number: SZY20150524). Female C57BL/6 mice were purchased from the SLAC Laboratory (Shanghai, China). Animals were housed and bred according to standardized procedures, under controlled temperature (22–23°C) and on a 12-h light, 12-h dark cycle. Six-week-old female mice were fed with HF diet for 12 weeks to induce obesity and these mice were then randomly divided into three groups according to body weight: Chow group (10% of calories derived from fat), HF group (HF, 60% of calories derived from fat), and Silymarin group (HF diet supplemented with silymarin powder, Legalon from MADAUS GmbH, Germany, at dose of 40 mg/100 g diet). Mice were treated for additional 6 weeks. Food intake amount was measured by recording food weight every 2 days throughout the experiment. The amount of food intake over a 24-h period was calculated.

### Intraperitoneal Glucose Tolerance and Insulin Tolerance

At the end of the treatment, mice were fasted overnight (12 h). The baseline glucose values (0 min), prior to the injection of glucose (1 g/kg body weight), were measured through tail vein. Additional blood samples were collected at regular intervals (15, 30, 60, and 90 min) during glucose tolerance tests. For the IPITT, non-fasted glucose levels were determined from the tail vein (0 min). Then insulin (Sigma, St. Louis, MO, USA) was injected intraperitoneally (0.75 U/kg body weight). Subsequent blood samples were taken at 15, 30, 60, 90, and 120 min after insulin administration for glucose measurement.

### Serum Chemistry Analysis

At the end of animal experiment study, mice were anesthetized 20% urethane (Sigma, St. Louis, MO, USA) and cardiac blood was taken. Levels of serum alanine aminotransferase, AST, TG, TC, HDL-c, and LDL-c were measured using a Hitachi 7020 Automatic Analyzer (Hitachi, Limited, Tokyo, Japan) with 100 μl of heart blood serum.

### Histochemistry

Liver tissues were fixed in formalin, and paraffin-embedded sections were cut at 5 μm. Sections were stained with haematoxylin and eosin according to a standard procedure.

### Hepatic Lipid Content Analysis

Lipid content was measured as described (Folch et al., 1957). Briefly, liver tissues (100 mg) were homogenized with 2 ml chloroform-methanol and then agitated overnight on an orbital shaker at 4°C. The homogenate was then centrifuged (5 min at 5,000 rpm), 0.9% NaCl solution was subsequently added to the liquid phase before the samples were vortexed. Phase separation was induced by centrifugation (2,000 rpm for 10 min), and the bottom phase was removed to a new tube and evaporated to dryness, Samples were then resuspended in 500 μl chloroform-1% Triton X-100, evaporated to dryness, and finally resuspended in 500 μl of water. The quantities of total cholesterol and triglycerides (KINGHA WK, China) in liver lipid extracts were then assayed by using enzymatic kits according to the manufacturers’ protocols.

### RNA Extraction and Quantitative PCR

Total RNA from HepG2 cells or mouse livers was isolated using the TRIzol method (Invitrogen, Carlsbad, CA, USA). The first-strand cDNA was synthesized with a cDNA synthesis kit (Fermentas, Madison, WI, USA). Quantitative real-time polymerase chain reaction (PCR) was carried out using SYBR green PCR Mastermix. The results were analyzed on an ABI StepOnePlus real-time PCR system (Applied Biosystems, USA). Values were normalized to Beta-actin. Sequences for primers are listed in **Tables 1** and **2**.

**Table 1:** Sequences of the human primers used in real time polymerase chain reaction (PCR).

Gene	Sense primer	Anti-sense primer
*NR1H4*	ATGGGAATGTTGGCTGAATG	CCTGCATGACTTTGTTGTCG
*NR0B2*	AGGCCTCCAAGCCGCCTCCCACATTGGGC	GCAGGCTGGTCGGAAACTTGAGGGT
*LRH1*	CTAGAAGCTGTAAGGGCCGACCG	TCCATTGGCTCGGATGAGGGCT
*SULT2A1*	AACAGGACACAGGAAGAACCAT	CAGTCCCCAGATACACCTTTTC
*ABCC2*	TCGGAATGTGAATAGCCTGAAG	CGCAAGGATGATGAAGAATATCG
*ABCB4*	GCAGACGGTGGCCCTGGTTGG	TGGAAAACAGCACCGGCTCCTG
*APOC2*	GAGATGCCTAGCCCGACCTTCCTCAC	GCTCAGTCTGAACCTGGGGGATCAGG
*CYP7A1*	GAGAAGGCAAACGGGTGAAC	AGCACAGCCCAGGTATGGA
*CYP8B1*	CCCTCTTTCCCTACCTCTCAGT	AAGTGTGTGACCATAAGCAGGA
*PPAR*α	GAAATGGGAAACATCCAAGAGA	CACAGGATAAGTCACCGAGGA
*ACOX1*	CCAAGCTTTCCTGCTCAGTGTT	CCCCCAGTCCCTTTTCTTCA
*CPT1*α	TGGCGTCTGAGAAGCATCAGCATA	ACACCACGTAAAGGCAGAAGAGGT
*ACC*	GGATGGTGTTCACTCGGTAATAGA	GGGTGATATGTGCTGCGTCAT
β*-ACTIN*	AATCTGGCACCACACCTTCTA	ATAGCACAGCCTGGATAGCAAC

**Table 2:** Sequences of the mouse primers used in real time PCR.

Gene	Sense primer	Anti-sense primer
β*-actin*	TGTCCACCTTCCAGCAGATGT	AGCTCAGTAACAGTCCGCCTAGA
*Nr1h4*	TTCCTCAAGTTCAGCCACAG	TCGCCTGAGTTCATAGATGC
*Nr0b2*	GGAGTCTTTCTGGAGCCTTG	ATCTGGGTTGAAGAGGATCG
*Lrh1*	TCAGTTCGATCAGCGGGAGTTTGT	TGCAGGTTCTCCAGGTTCTTCACA
*Hnf4*α	GTGCTTCCGGGCTGGCATGAA	AGGTGATCTGCTGGGACAGAACC
*Ppar*α	AGGCTGTAAGGGCTTCTTTCG	GGCATTTGTTCCGGTTCTTC
*Ppar*β	AGTGACCTGGCGCTCTTCAT	CGCAGAATGGTGTCCTGGAT
*Ppar*γ	CGCTGATGCACTGCCTATGA	AGAGGTCCACAGAGCTGATTCC
*Pgc1*α	TGTTCCCGATCACCATATTCC	GGTGTCTGTAGTGGCTTGATTC
*Pgc1*β	GGGTGCGCCTCCAAGTG	TCTACAGACAGAAGATGTTATGTGAACAC
*G6pc*	GTGGCAGTGGTCGGAGACT	ACGGGCGTTGTCCAAAC
*Pck1*	CACCATCACCTCCTGGAAGA	GGGTGCAGAATCTCGAGTTG
*Apoc2*	TGATGTTGGGAAATGAGG	ATCGGGTATGTCTTCTGGTA
*Apoai*	ACTCGGGACTTCTGGGATA	AGTGTCTTCAGGTGGGTTTT
*Acc*	GAATCTCCTGGTGACAATGCTTATT	GGTCTTGCTGAGTTGGGTTAGCT
*Scd1*	CTTATCATTGCCAACACCA	CTTCTCGGCTTTCAGGTC
*Tnf*α	ATGGATCTCAAAGACAACCAACTAG	ACGGCAGAGAGGAGGTTGACTT
*Mcp-1*	AGGTCCCTGTCATGCTTC	GTGCTTGAGGTGGTTGTG
*Il1*β	TCGTGCTGTCGGACCCATAT	GGTTCTCCTTGTACAAAGCTCATG
*Cox2*	TGAGCAACTATTCCAAACCAGC	GCACGTAGTCTTCGATCACTATC
*Cyp7a1*	TGATCCTCTGGGCATCTCAAGCAA	AGCTCTTGGCCAGCACTCTGTAAT
*Cyp8b1*	GGACAGCCTATCCTTGGTGA	GACGGAACTTCCTGAACAGC
*Cyp27a1*	GAGAGTGAATCAGGGGACCA	CCATTTGGGAAGGAAAGTGA
*Ntcp*	TATCAGCCCCCTTCAATTTC	GTGAGCCTTGATCTTGCTGA
*Baat*	CCATTGAAAAAGCTCATGGA	ATCAGCTGTGCTATGGCTTG
*Bacs*	GATGCCCTTGCTACACTCT	AGGACAAGCCCTATCGTAT
*Abcb11*	CGGACCTGTATTGTCATTGC	CCCTTCTGGTCCATCAGTTT

### Quantification of BAs in Mice

Measurement of TBAs in mouse livers and feces was performed as the following. Livers were weighed and homogenized in 10X volume water. TBA level determination was performed on homogenate samples using the TBA Measurement Kit (KeHua, China) after quantification of intracellular protein concentration. The TBA levels were normalized to intracellular protein content. Protein concentrations were determined by BCA protein assay kit (Sigma, St. Louis, MO, USA). The fecal TBA analysis was performed as the same method described above and calculated by normalizing the feces weight.

To measure hepatic BA pool size and composition, liver-tissue samples were weighed, and TBA was extracted in 5X volumes of acetonitrile followed by centrifugation at 14,300 rpm for 10 min. The supernatant was dried under nitrogen steam before re-dissolved in methanol solution (methanol:water:formic acid = 50:50:0.01) and then subject to centrifugation at 14,300 rpm for 10 min. Supernatant bile salt species were analyzed by ultra-performance liquid chromatography (UPLC) triple time of flight/MS analysis (UPLC-MS, Waters Co., MA, USA). The relevant parameters were described in a previous publication (Yang et al., 2012).

### Molecular Docking

The crystal structure of NR FXR (PDB code 1OT7) was retrieved from the Research Collaboratory for Structural Bioinformatics (RCSB) protein data bank. Silybin A and silybin B were constructed using the sketcher module in Sybyl and their minimum energy conformations were calculated using the Minimize module of Sybyl. The force field was Tripos with an 8 Å cutoff for non-bonded interactions, and the atomic point charges were also calculated with Gasteiger-Huckel. Minimizations were achieved using the steepest descent method for the first 100 steps, followed by the Broyden-Fletcher-Goldfarb-Shanno (BFGS) method until the root-mean-square (RMS) of the gradient became <0.005 kcal/(mol⋅Å). The Surflex-Dock module implemented in the Sybyl program was used for the docking studies. The obeticholic acid binding pocket of FXR was used for the docking simulation. Both silybin A and silybin B were docked into the binding site by an empirical scoring function and a patented search engine in Surflex-Dock, applied with the automatic docking. Other parameters were established by default in the software.

### Statistical Analysis

All values were expressed as means ± SEM and analyzed using the statistical package for the social science (SPSS, version 15.0). Paired or unpaired two tailed *t*-tests were used to detect difference in the mean values of treatment group and control and analysis of variance (ANOVA) for the difference among more than two groups. Differences with *P* values <0.05 were considered to be statistically significant.

## Results

### Silymarin and Silybin Stimulate FXR Transactivity *In vitro*

**FIGURE 1:**
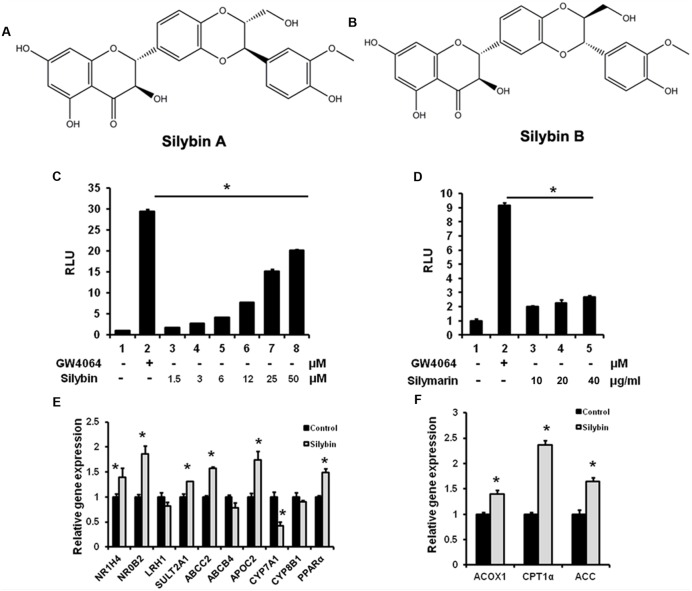
**Silybin and silymarin activate FXR transactivity *in vitro*. (A,B)** Structure of Silybin A and Silybin B. Silybin **(C)** and silymarin **(D)** activate FXR transactivity. HEK293T cells were co-transfected with phFXR, phRXR expression plasmids, and FXR-dependent reporter (EcRE-LUC) for 24 h and treated with the FXR agonist GW4064 (10 μM), control (DMSO), silybin (1.5, 3, 6, 12, 25, 50 μM) and silymarin (10, 20, and 40 μg/ml) for another 24 h. The relative luciferase activities (RLU) were measured by comparison to renilla luciferase activities. **(E,F)** The relative gene expression levels in silybin-treated (25 μM) and control (DMSO) HepG2 cells. *Beta-ACTIN* was used as an internal control for normalizing the mRNA levels. The results represent three independent experiments, and data are presented as means ± SEM. (*n* = 3). ^∗^*P* < 0.05 vs. HEK293T or HepG2 control.

To test whether silymarin and its major constituent silybin (silybin A:B = 1:1, **Figures 1A,B**) are able to activate FXR, we performed a gene reporter assay by co-transfected hFXR, hRXR expression vectors, and FXR-dependent reporter (EcRE-LUC) into HEK293T cells. The results demonstrated that silybin activated the FXR transactivity in a dose-dependent manner (**Figure 1C**). Silymarin showed similar effects but with lower activity in (**Figure 1D**). We also analyzed the effects of silybin and silymarin on other important NR transactivity and the results showed that there were no effects of silybin or silymarin on PXR, PPARγ, α, β/δ, and LXRα, β transactivities (data not shown), suggesting that the silybin activates FXR specifically.

Farnesyl X receptor agonists have been shown to activate a series of target genes. We therefore examined whether silybin could regulate known target gene expression in HepG2 cells. As expected, silybin treatment induced FXR target gene expression when compared with mock control groups (**Figures 1E,F**), suggesting an increased FXR transactivity by silybin.

TGR5, abile acid sensitive G-protein coupled receptor, has been reported to bind with diverse FXR agonists (Chen et al., 2011; Keitel and Häussinger, 2012). To clarify whether silybin and silymarin also activate TGR5-cAMP signaling, we transiently transfected hTGR5 expression plasmid and pCRE-Luc reporter into HEK293T cells over a period of 24 h. Even with a high dose (Silybin at 50 μM and silymarin at 40 μg/ml), neither silybin nor silymarin stimulated the pCRE-Luc reporter signaling (data not shown), indicating that both drugs have limited influence on activating hTGR5.

### Silybin Interacts with FXR

**FIGURE 2:**
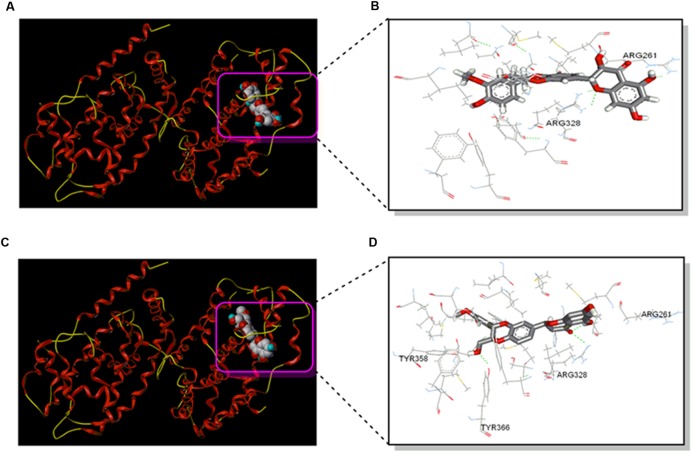
**Silybin interacts with FXR. (A)** The structure of the complex of the FXR and Silybin A by molecular docking. **(B)** The interaction map of the complex of the FXR and silybin A in the binding site. **(C)** The structure of the complex of the FXR and silybin B by molecular docking. **(D)** The interaction map of the complex of the FXR and silybin B in the binding site.

To further test potential direct interaction between silybin and NR FXR, the structure of the complex of FXR and silybin was analyzed by molecular docking. The obeticholic acid binding site of FXR has been validated as the binding pocket of silybin. Both silybin A and silybin B were docked into the binding pocket, the pose that ranked first complex with NR FXR were shown in **Figures 2A,C**. Silybin skeleton was surrounded by a hydrophobic pocket composed of Arg261, Met262, Leu284, Met287, Ala288, His291, Ile294, Met325, Arg328, Ser329, Ile332, Phe333, Leu345, Ile349, Phe363, and Tyr366, where van der Waals forces play a dominant role in the interactions. These interactions were postulated to be the primary reason for silybin activity. The specific interaction of silybin A and silybin B with FXR is also displayed in **Figures 2B,D**. Hydrogen-bonding interactions showed that silybin A was bound to Arg261 and Arg328 of subunit A of FXR, while silybin B is bound to Arg328 and Tyr366 of subunit A of FXR. These H-bonds help to enhance the binding stability.

### Silymarin Exerts Hypoglycaemic Effects in DIO Mice

**FIGURE 3:**
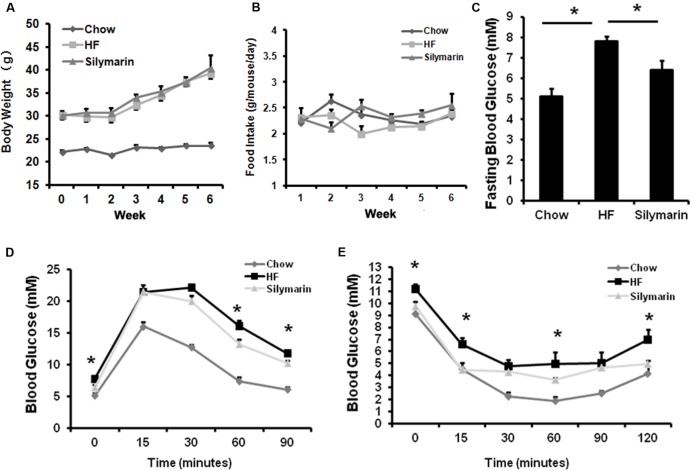
**Silymarin improves blood glucose homeostasis in DIO C57BL/6 mice. (A)** Body weight during the 6-week treatment. **(B)** Food intake amount. **(C)** Fasting blood glucose level. **(D)** Glucose tolerance test (GTT). The mice were fasted overnight, and glucose were intraperitoneally injected (1 g of/kg body weight) and blood glucose levels were determined at the indicated time points. **(E)** Insulin tolerance test (ITT). The non-fasted mice were intraperitoneally injected with insulin (0.75 U/kg body weight) and blood glucose levels were determined. Data are presented as means ± SEM (*n* = 7). ^∗^*P* < 0.05, vs. Chow or HF group, NS, No significance.

To test whether silymarin affects glucose homeostasis, obese C57BL/6 mice fed HFD were divided in two groups, one continued to feed on a HF diet as control and the other fed HF diet mixed with silymarin (100 mg Legalon or 40 mg silymarin/100 g diet) for additional 6 weeks. Silymarin treatment significantly ameliorated fasting glucose (**Figure 3C**), glucose intolerance at 30 and 60 min (**Figure 3D**), and insulin tolerance in obese mice at 15, 60, and 120 min (**Figure 3E**) without changes in body weight gain or food intake (**Figures 3A,B**), suggesting that silymarin improves glucose homeostasis in DIO mice.

### Silymarin Attenuates Dyslipidaemia in DIO Mice

**FIGURE 4:**
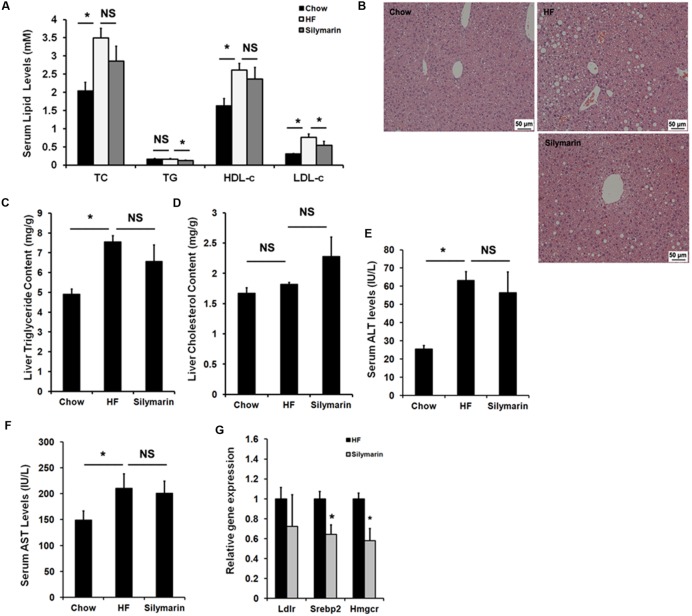
**Silymarin ameliorates dyslipidemia in DIO mice. (A)** Serum TC, TG, LDL-c, HDL-c. **(B)** H&E staining of liver sections (×200). **(C)** Liver TG level. **(D)** Liver TC level. **(E)** Serum ALT level. **(F)** Serum AST level. **(G)** The relative gene expression levels in the livers. *Beta-Actin* was used as an internal control for normalizing the mRNA levels. Data are presented as means ± SEM (*n* = 7). ^∗^*P* < 0.05, vs. Chow or HF group, NS, No significance.

We next assayed the lipid profile in the diet-induced obese mice. As expected, HF diet feeding elevated serum TC, HDL-c, and LDL-c levels in HF-fed control mice compared to Chow controls (**Figure 4A**), and notably, 6-week silymarin treatment decreased serum LDL-c levels in DIO mice (**Figure 4A**), but not TG, TC, and HDL-c. OCA and other FXR agonists have been identified to have therapeutic effects against NFALD and NASH. We therefore determined whether silymarin could improve hepatic steatosis. The HE staining results showed that silymarin treatment did not significantly reduce hepatic steatosis (**Figure 4B**), and this was confirmed by lipid content measurement, which showed no notably reduction when compared to HF-fed controls (**Figures 4C,D**). Serum ALT and AST levels were markedly higher in DIO mice compared to control mice, but no difference in ALT or AST levels were found between DIO controls and silymarin-treated mice (**Figures 4E,F**). Subsequent gene expression analysis (**Figure 4G**) indicated that silymarin markedly decreased mRNAs of *Srebp2* and *Hmgcr*, two genes involved in cholesterol metabolism in DIO mice. Collectively, these results suggest that silymarin corrects the serum dyslipidaemia in DIO mice.

### Silymarin Alters BA Composition *In vivo*

**FIGURE 5:**
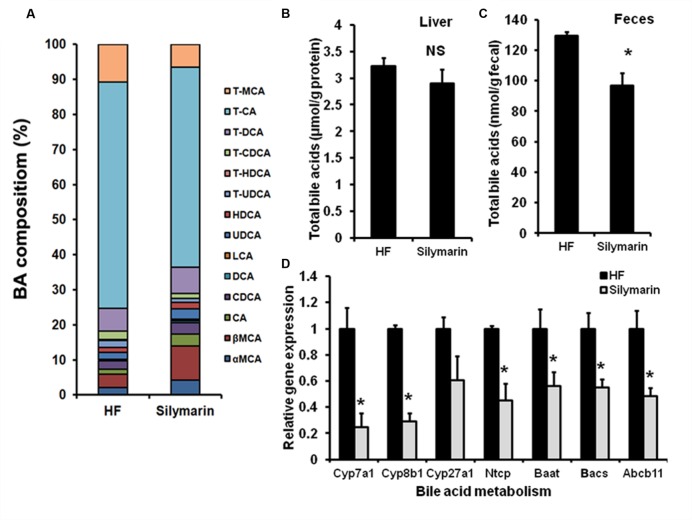
**Silymarin alters BA composition in DIO mice. (A)** Hepatic TBA level. **(B)** Fecal TBA level. **(C)** Hepatic BA composition. **(D)** The relative gene expression levels in the livers. *Beta-Actin* was used as an internal control for normalizing the mRNA levels. Data are presented as means ± SEM (*n* = 7). ^∗^*P* < 0.05, vs. HF group, NS, No significance.

The central function of FXR is to regulate BA homeostasis. Therefore, TBAs in livers and feces were determined following analysis of hepatic BA composition. After silymarin treatment, DIO mice showed markedly decreased TBA levels in feces and a tendency, although not statistically significant, toward a decreased level in liver, compared with HF controls (**Figures 5B,C**). Meanwhile, the composition of hepatic BA was also altered by silymarin treating. In contrast to high fat diet fed mice, silymarin treatment strikingly decreased fraction of TCA, murine taurocholic acid (mTCA), TCDCA, and TUDCA, which was accompanied with an increasing fraction of the α,β murine taurocholic acid (α,β mTCA), and CA in silymarin-treated DIO mice (**Figure 5A**; **Table 3**). Moreover, gene expression experiments showed that a series of genes such as *Cyp7a1*, *Cyp8b1*, *Ntcp*, *Baat*, *Bacs*, and *Abcb11* were markedly decreased in the silymarin treatment group as compared to HF controls (**Figure 5D**). These data suggested silymarin contributes to the changes of BA metabolism via influencing the BA signal pathway.

**Table 3:** Hepatic BAs composition (%) in DIO mice.

BA	HF	Silymarin
T-MCA	11.24	6.22
T-CA	67.32	54.91
T-DCA	6.74	7.25
T-CDCA	2.54	1.31
T-HDCA	0.38	0.09
T-UDCA	2.02	1.07
HDCA	1.39	1.75
UDCA	2.18	2.87
LCA	0.05	0.35
DCA	0.35	0.53
CDCA	2.44	3.22
CA	1.46	3.13
βMCA	4.04	9.43
αMCA	2.17	4.07

### Silymarin Mediates the Hepatic FXR Signaling and Alleviates Inflammation

**FIGURE 6:**
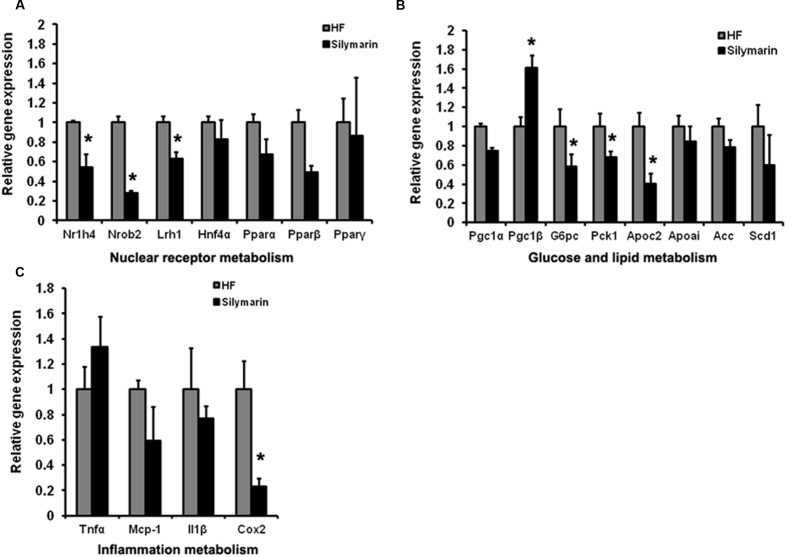
**Silymarin regulates FXR signal and alleviates the inflammation in DIO mice liver.** The mRNA levels of NRs **(A)**, glucose and lipid metabolism related genes **(B)**, and inflammation metabolism related genes **(C)** in the livers. *Beta-actin* was used as an internal control for normalizing the mRNA levels. Data are presented as means ± SEM (*n* = 7). ^∗^*P* < 0.05, vs. HF group.

As a major organ tissue delivering FXR signals, the liver is involved in diverse metabolic physiological functions as well as the regulation of inflammation. We further address whether silymarin mediates signaling downstream of FXR in the liver. We compared the expression levels of a cluster of FXR target genes in the liver tissues between HF control mice and HF supplemented silymarin group mice using quantitative RT-PCR. Liver samples from silymarin-treated mice displayed significantly lower expression levels of *Nr1h4*, *Nr0b2*, *Lrh1*, *G6pc*, *Pck1*, and *Apoc2* as well as inflammatory cytokine *Cox2* while *Pgc1*β levels were significantly higher (**Figures 6A–C**). These results suggest that silymarin is able to alter hepatic FXR signaling and therefore modulates its downstream targets related to gluconeogenesis, lipogenesis, and inflammation in DIO mouse livers.

### Silymarin and Silybin Inhibit NF-κB Signaling Associated with FXR Activation

**FIGURE 7:**
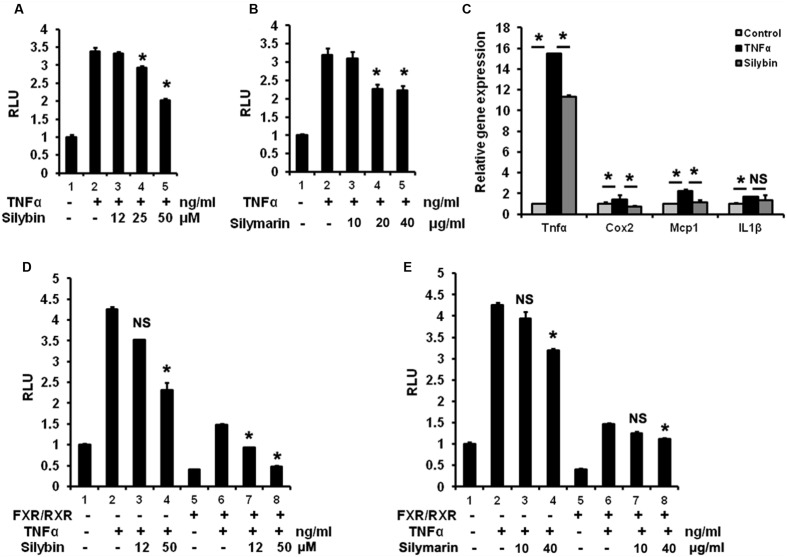
**Silymarin and silybin inhibit NF-κB signaling via activating FXR.** Silybin **(A)** and silymarin **(B)** repressed NF-κB transactivity in HepG2 cells. HepG2 cells were co-transfected with the NF-κB reporter plasmids and P65 expression plasmids for 24 h and pretreated with the control (DMSO), silybin (12, 25, 50 μM)or silymarin (10, 20, and 40 μg/ml) for 18 h before treatment with TNFα (10 ng/mL) for another 6 h. The RLU were measured by comparison to renilla luciferase activities. ^∗^*P* < 0.05 compared to the groups of TNFα (*n* = 3). **(C)** The relative gene expression levels in silybin-treated (25 μM) and control (DMSO) HepG2 cells incubated with TNFα (10 ng/mL). *Beta-actin* was used as an internal control for normalizing the mRNA levels. ^∗^*P* < 0.05 compared to the groups of TNFα or controls (*n* = 3). Silybin **(D)** and silymarin **(E)** enhanced the inhibition of NF-κB signaling via activating FXR. HepG2 cells were co-transfected with the NF-κB reporter plasmids and P65 expression plasmids with or without FXR/RXR expression plasmids before treatment with control (DMSO), TNFα (10 ng/mL), silybin (12 and 50 μM), or silymarin (10 and 40 μg/ml) according to **(A,B)**. RLU were measured by comparison to renilla luciferase activities. ^∗^*P* < 0.05 vs. the groups of TNFα (*n* = 3). The results represent three independent experiments, and data are presented as means ± SEM. NS, No significance.

To test potential inhibitory effect on NF-κB pathway by silybin and silymarin *in vitro*, HepG2 cells over-expressing p65 and NF-κBx3-Luc were pre-treated with silybin and silymarin at the indicated different concentrates, in a condition with an enhanced NF-κB reporter activity by TNFα stimulation. Our results (**Figures 7A,B**) showed that silybin (25 and 50 μM) and silymarin (20 and 40 μg/ml) suppressed the NF-κB transactivity induced by TNFα in HepG2 cells. Silybin (25 μM) significantly reduced the levels of TNFα-induced expression of *TNF*α, *COX2*, and *MCP-1* mRNA as compared to vehicle (**Figure 7C**). FXR agonists such as GW4064 and 6ECDCA were reported to inhibit NF-κB activity via FXR activation (Wang et al., 2008). To test whether silybin and silymarin have similar effects, we pre-treated HepG2 cells with silybin and silymarin prior to TNFα treatments. Silybin (50 μM) and silymarin (40 μg/ml) weakened NF-κB signaling in the condition with p65/NF-κBx3-Luc transfection alone. Meanwhile, the transfection of these cells with p65/NF-κBx3-Luc plus FXR/RXR inhibited NF-κB activity in the absence of exogenous drug (**Figures 7D,E**). Furthermore, addition of silybin to HepG2 cells with FXR/RXR overexpression showed an augmented suppressing effects, resulting in significant depression on NF-κB activity (**Figures 7D,E**). These results indicate that silybin and silymarin may strengthen interference with NF-κB activity correlated with the activation of FXR.

## Discussion

In the present study, we provided evidence showing that silybin and silymarin activated NR FXR and triggered a negatively crosstalk with NF-κB. We also confirmed that silymarin can be used to treat MS, especially to ameliorate hyperglycaemia, dyslipidaemia, and regulate BA homeostasis in DIO mice. These effects were probably achieved via the stimulation of FXR signaling and suppression of NF-κB responses. Our data support the possibility to use silybin as a novel natural FXR agonist that can effectively treat and cure MD.

Previous studies have confirmed that silybin represents about 50–70% bioactive compounds in the silymarin extract (Kim et al., 2003; Loguercio and Festi, 2011). FXR is an important molecular target that can be used to control hepatobiliary diseases of enterohepatic cycling, which has prompted the idea that silybin might be correlated with FXR (Matsubara et al., 2013). As expected, FXR gene reporter assay revealed that silybin and silymarin apparently activated FXR reporter signal in a dose dependent manner, which was proved by the change of mRNA levels of FXR downstream target genes in HepG2 cells, including increased expressions of *NR0B2*, *SULT2A1*, *ABCC2*, *APOC2*, and *PPAR*α, but decreased expression of *CYP7A1*. The molecular docking assay further supported a direct binding between silybin and FXR. These data support that silybin and silymarin are capable of enhancing the FXR transactivity. These results suggest that silybin is a potential agonist of FXR.

Farnesyl X receptor activation leading to improving lipid and glucose metabolism has received a particularly intense attention. Fasted FXR-null mice exhibit impaired glucose intolerance, insulin insensitivity, and increased levels of plasma triglyceride, free fatty acids, and cholesterol (Sinal et al., 2000; Zhang et al., 2006; Cipriani et al., 2010; Prawitt et al., 2011). Treatment with FXR agonists, GW4064, or INT-747, has been shown to reduce plasma triglyceride, glucose levels, and improve insulin sensitivity in several models of obesity and diabetes (Zhang et al., 2006; Mason et al., 2010). These phenotypes have been attributed to the ability of FXR to control the expression of several genes involved in glycolipid metabolism. In our study, we also found that silymarin treatment effectively lowered HF diet-induced hyperglycaemia and insulin resistance in DIO mice, similarly to the previous reports (Kazazis et al., 2014; Voroneanu et al., 2016). Furthermore, gene expression analysis has demonstrated down-regulation in the expression of certain genes, including *Pgc1*α, *G6pc*, and *Pck1*, in silymarin-treated mice, suggesting effective inhibition of hepatic gluconeogenesis, which could explain the anti-diabetic efficacy of silymarin. Interestingly, these gene changes were reminiscent of recent findings that FXR activation downregulates the gluconeogenic program.

Previous reports showed that silymarin may be able to alleviate hepatic steatosis (Ni and Wang, 2016). However, we did not observe an improvement of the morphology in the liver of DIO mice. This probably caused by the shorter phase of treatment in our study. Extension of the treatment may result in better result in morphology of the liver tissues.

It is well established that the inflammation response is positively correlated with IR and diabetes (Baker et al., 2011). Recently, silybin and silymarin were repeatedly reported to be able to block inflammatory reactions in multifarious cells via antagonizing transcription factor NF-κB (Bremner and Heinrich, 2002; Dhanalakshmi et al., 2002; Salamone et al., 2012). Consistently, we obtained similar results showing that silybin and silymarin inhibited the TNFα-induced NF-κB signal in HepG2 cells as well as reducing *TNF*α, *COX2*, and *MCP1* expression levels stimulated by TNFα in human hepatocytes. These inflammatory cytokines are all shown to be directly regulated by NF-κB (Baker et al., 2011). Notably, it is unknown whether silybin interacts with NF-κB, i.e., the mechanism underlying silybin control of NF-κB remains to be elucidated. The anti-inflammatory effects of FXR have been well documented (Shaik et al., 2015). An FXR agonist has been reported to activate anti-inflammation responses via negatively regulation of NF-κB (Wang et al., 2008). Deficiency of FXR was also found to increase the development of liver and intestine cancer via the upregulation of inflammation (Yang et al., 2007). In the present study, we found that NF-κB activity was largely inhibited in HepG2 cells transfected with FXR/RXR in the absence of xenobiotics. We found that silybin treatment, significantly enhanced the inhibitory effect on NF-κB activitys, compared to the treatment condition without FXR/RXR. Silybin suppressed NF-κB transactivity without FXR/RXR transfection. Furthermore, the inhibitory efficiency of silybin on the NF-κB reporter was increased by nearly 20% in HepG2 cells with FXR/RXR overexpression. This suggests that suppression of NF-κB transactivity by silybin may be correlated with the activation level of FXR signaling. That silymarin treatment notably lowered *Cox2* gene expression in the liver of DIO mice also supported the anti-inflammation action of silymarin *in vivo*. Our data suggested that reduced hepatic inflammation signal in DIO mice by silymarin may contribute to the antidiabetic effect.

Farnesyl X receptor is a key modulator of BA metabolism in the enterohepatic system that acts via various feedforward and feedback loops. FXR deficiency impairs BA homeostasis (Matsubara et al., 2013). After a 6-week treatment, the hepatic and fecal TBA levels in silymarin-treated mice were lower than in HF controls, indicating a decreased BA pool size. In addition, decreased proportions of TCA, mTCA, TCDCA, and TUDCA with increased fraction of (α, β mTCA) and CA in silymarin-treated mice compared to HF controls showed an altered hepatic BA composition. Hepatic *Lrh1*, *Ntcp*, *Cyp7a1*, and *Cyp8b1* are essential genes in enterohepatic BA metabolism and negatively regulated by FXR activation, which respectively, promotes BA gene expression, uptakes of conjugated BAs, controls BA synthesis and BA hydroxylation. Consistent with the characteristics of reported FXR agonists, silymarin markedly reduced *Lrh1*, *Ntcp*, *Cyp7a1*, and *Cyp8b1* gene expression levels in DIO mice, indicating that FXR is able to activate the feedback loop of FXR signaling in liver tissue of the mice. This finding further supports that silymarin is a natural FXR agonist modulating BA homeostasis.

One complicating factor in the study of FXR biology is that BAs are also ligands for multiple other receptors controlling MS (Sinal et al., 2000). Here we excluded the agonist activity of silybin on activating several important BA-related receptors such as PPARα, γ, LXRα, β, PXR, and TGR5 *in vitro* via reporter assays. However, we found that silymarin elevated the proportions of LCA, (α, β mTCA), and CA *in vivo*. All of these compounds are endogenous ligands for TGR5, which indicates that endogenous TGR5 activation by several BAs might mediate partial silymarin therapeutic effects.

## Conclusion

we found that both silybin and silymarin stimulated FXR transactivity and inhibited NF-κB signaling *in vitro*. Silmarin supplementation ameliorated insulin resistance, hyperlipidaemia and inflammation progress, and reconstituted the BA pool in DIO mice. Our data suggest that the effects of silymarin on metabolic disorders may be related to FXR activation and NF-κB inhibition and that the majority of these effects are mediated by its main active compound, silybin. Whether FXR is involved in the treatment of silymarin for toxic liver damage, hepatitis, and cirrhosis warrants further studies.

## Author Contributions

MG, Y-MM, LY, GJ, QT, and CH conceived the experiments. MG, PZ, YZ, YW, SF, YnL, and YfL. performed the experiments, JH performed molecular docking assay, MG and CH analyzed the data. All authors discussed the results and commented on the manuscript. MG, QT, and CH wrote the manuscript.

## Conflict of Interest Statement

The authors declare that the research was conducted in the absence of any commercial or financial relationships that could be construed as a potential conflict of interest.

## Abbreviations

ALT: alanine aminotransferase
AST: aspartate transaminase
BA: bile acid
CA: cholic acid
DIO: diet-induced obesity
DMSO: dimethylsulfoxide
FXR: farnesyl X receptor
HDL-c: high density lipoprotein cholesterol
HF: high-fat
IPITT: intraperitoneal insulin tolerance test
LDL-c: low density lipoprotein cholesterol
NAFLD: non-alcoholic fatty liver disease
NF-κB: nuclear factor kappa B
NR: nuclear receptor
MS: metabolic syndrome
Rosi: rosiglitazone
TBA: total bile acid
TC: total cholesterol
TCA: taurocholic acid
TCDCA: tauro-chenodeoxycholic acid
TG: triglyceride
TUDCA: tauro-ursodeoxycholic acid
